# Serum docosahexaenoic acid as a predictor of hospital readmission in chronic obstructive pulmonary disease exacerbation: a retrospective cohort study

**DOI:** 10.7717/peerj.20865

**Published:** 2026-02-19

**Authors:** Qiqiang Zhou, Yating Wang, Chun Chang, Xiaoyan Gai, Yahong Chen, Ying Liang, Yongchang Sun

**Affiliations:** 1 Department of Respiratory and Critical Care Medicine, Peking University Third Hospital, Beijing, China; 2 Research Center for Chronic Airway Diseases, Peking University Health Science Center, Beijing, China

**Keywords:** Chronic obstructive pulmonary disease, Exacerbation, Omega-3 fatty acids, Docosahexaenoic acid

## Abstract

**Background:**

The beneficial effects of omega-3 fatty acids for patients with chronic obstructive pulmonary disease (COPD) had been observed, including attenuating lung function decline and reducing their respiratory symptom burdens. However, the impact of omega-3 fatty acids on COPD exacerbation-related outcomes remains unclear. This study aimed to evaluate whether reduced serum omega-3 fatty acid levels are associated with a higher risk of future hospital readmission due to COPD exacerbation (ECOPD).

**Methods:**

This retrospective cohort study included 88 patients hospitalized for ECOPD between April 2017 and March 2018. Clinical data were collected, and serum omega-3 fatty acid levels were analyzed using liquid chromatography-mass spectrometry (LC-MS). All patients were followed up for a median period of 53.5 months and categorized into two groups based on whether they experienced ECOPD-related readmission during the follow-up period. The clinical characteristics and serum levels of omega-3 fatty acid levels, including docosahexaenoic acid (DHA) and eicosapentaenoic acid (EPA), between the two groups were compared. Additionally, patients were categorized into low and high DHA groups based on the median DHA level, and the association between DHA level and ECOPD-related readmission rate was analyzed using a Cox regression model.

**Result:**

Patients who experienced ECOPD-related readmission during the follow-up period (*n* = 36) had lower serum levels of DHA than those who did not experience readmission. The serum levels of EPA did not significantly differ between groups. Kaplan–Meier curve showed that patients in the low-DHA group exhibited a significantly higher ECOPD-related readmission rate compared to those in the high-DHA group (log-rank *p* = 0.023). Multivariable Cox regression analysis identified low DHA level as an independent risk factor for ECOPD-related readmission. A nomogram based on DHA levels demonstrated good predictive performance.

**Conclusion:**

A low DHA level serves as an independent risk factor for ECOPD-related readmission, suggesting DHA may have a potential protective effect to reduce the risk of exacerbation in patients with COPD.

## Introduction

Chronic obstructive pulmonary disease (COPD) is a chronic inflammatory airway disease characterized by persistent airflow limitation, posing a significant social and economic burden on global public health (Global Initiative for Chronic Obstructive Lung Disease, 2025). Exacerbations of COPD (ECOPD) are significant adverse events in the course of disease, which not only severely impacts patients’ health, but is also closely associated with hospitalization rates, readmission rates, and disease progression (Seemungal et al., 1998; Wedzicha & Seemungal, 2007). In particular, patients experienced hospitalization due to ECOPD have a worse long-term prognosis, with a five-year all-cause mortality rate as high as 50% (Hoogendoorn et al., 2011). The clinical trajectory of ECOPD exhibits marked heterogeneity in symptom presentation, therapeutic response, and prognostic outcomes, underscoring the urgent need for reliable predictive biomarkers. Currently, there is a lack of specific biomarkers for predicting future admission risk due to ECOPD. Identifying potential indicators for predicting admission risk is crucial for improving patient management and optimizing treatment strategies.

In recent years, omega-3 fatty acids have garnered significant attention for their anti-inflammatory effects in COPD pathogenesis. Docosahexaenoic acid (DHA) and eicosapentaenoic acid (EPA) are the principal members of the omega-3 fatty acid family. Resolvin D1, which is derived from DHA, can mitigate smoking-induced emphysema and alveolar enlargement by reducing inflammation and oxidative stress levels (Pochettino et al., 2000). Maresin-1, another anti-inflammatory mediator derived from DHA, can reduce the infiltration of neutrophils in the airways, lower the levels of IL-6, TNF-α, and IL-8, and decrease the expression of the intercellular cell adhesion molecule-1 (ICAM-1), thereby downregulating the level of airway inflammation (Nordgren et al., 2015). Studies based on the general population and patients with COPD have demonstrated that higher omega-3 fatty acid intake in daily diet may lower the risk of developing COPD (Shahar et al., 2008; Varraso et al., 2015). Additionally, compared to those without COPD, individuals with COPD had decreased levels of omega-3 fatty acids (Varraso et al., 2015). A recent large study based on the general population found that for every 1% increase in plasma DHA levels, the decline rates of forced expiratory volume in one second (FEV1) and forced vital capacity (FVC) were reduced by 1.4 mL/year and 2.0 mL/year, respectively, and the incidence of airflow obstruction was decreased by 7% (Patchen et al., 2023). Additionally, Resolvin D1 has been shown to be associated with recovery from non-viral COPD exacerbations and to reduce levels of proinflammatory mediators such as interleukin-6 (IL-6) and C-X-C motif chemokine ligand 8 (CXCL8) in COPD-bronchial epithelial cells (COPD-BECs) (Finney et al., 2025), suggesting its potential role in modulating acute inflammatory responses during ECOPD episodes. These findings collectively demonstrate the beneficial role of omega-3 fatty acids in COPD. Compared with EPA and its downstream derivatives, DHA and its derivatives appear to be more strongly supported by evidence in terms of their roles in resolving inflammation, particularly in the context of COPD. Nevertheless, even though the protective role of omega-3 fatty acids, particularly DHA, in COPD has been well established, the previous studies have primarily focused on stable COPD. The impact of omega-3 fatty acids on acute exacerbation-related outcomes, such as hospital readmissions, remains unclear. Therefore, this study aimed to investigate whether serum omega-3 fatty acid levels, particularly DHA, in patients with COPD were associated with their future risk of readmission due to ECOPD. We hypothesized that DHA may be a protective factor for patients with COPD in reducing exacerbation-related readmission.

## Materials and Methods

This study was conducted in the Department of Respiratory and Critical Care Medicine at Peking University Third Hospital between April 2017 and March 2018. During this period, we consecutively enrolled 88 patients with ECOPD who were hospitalized in the wards of the department. All patients met the Global Initiative for Chronic Obstructive Lung Disease (GOLD) criteria for diagnosis of ECOPD characterized by worsening respiratory symptoms including dyspnea and/or cough and purulent sputum. The exclusion criteria included: the presence of other airflow-limiting diseases rather than COPD, concurrent pneumonia or active pulmonary tuberculosis, severe hepatic or renal insufficiency, malignancies, immunosuppressive conditions caused by chemotherapy or HIV infection, administration of systemic corticosteroids at any time within the past 4 weeks, and patients experiencing severe trauma or stress reactions. All patient care was aligned with current GOLD guidelines. Nebulized bronchodilators, as well as systemic corticosteroids or nebulized inhaled corticosteroids, were administered, and antibiotics were prescribed when standard indications were met, including: (1) the simultaneous presence of all three cardinal symptoms—worsening dyspnea, increased sputum volume, and purulent sputum; (2) the presence of any two of these symptoms, with purulent sputum being one of them; or (3) the need for invasive or non-invasive mechanical ventilation. Details of the sample size estimation using the PASS software (PASS 2025, version 25.0.2.) were provided in the Article S1.

Baseline clinical information was collected from the hospital’s electronic medical record system. The collected clinical data included demographic characteristics, smoking status, comorbidities, and the presence of hypercapnic respiratory failure, length of hospital stay (LOS) were required. Hypercapnic respiratory failure was defined as a partial pressure of arterial carbon dioxide (PaCO_2_) > 50 mmHg on arterial blood gas (ABG) analysis. Pulmonary function was assessed using spirometry, and FEV_1_% predicted and post-bronchodilator FEV_1_/FVC ratio were recorded for all patients. We recorded the comorbid conditions of each patient, including hypertension, cardiovascular disease, diabetes, cerebrovascular disease, chronic kidney disease, connective tissue diseases, among others. The overall comorbidity burden was assessed using the age-adjusted Charlson Comorbidity Index (ACCI) (Charlson et al., 1994). All enrolled patients were followed from discharge after the index hospitalization for ECOPD. For the patients experienced re-hospitalization due to re-exacerbation of COPD, the follow-up period was defined as the interval from the date of discharge after the index hospitalization to the date of the next hospitalization for ECOPD. Patients who did not experience ECOPD re-hospitalization would be followed up continuously until May 2023. Follow-up duration was reported in months, with a median of 53.5 months (interquartile range, 15.75–63.0 months).

Blood samples were collected within 24 h in the present hospital admission after at least 8 h of fasting. Routine blood tests and biochemical analyses were conducted in the hospital’s clinical laboratory. Laboratory measurements recorded in our study included peripheral blood cell counts and differentiation, C-reactive protein (CRP), D-dimer, fibrinogen, and other relevant markers.

Written informed consent was obtained from all participants or their legal guardians. The study was conducted in strict accordance with the ethical principles outlined in the 1964 Declaration of Helsinki and was approved by the Ethics Committee of Peking University Third Hospital (Approval No. M2017054; approval date: February 16, 2017).

### Liquid chromatography-mass spectrometry analysis

The methods for serum sample collection, lipid metabolite extraction, and liquid chromatography-mass spectrometry (LC-MS) analysis in this study were identical to those described in our previous research (Zhou et al., 2024). Briefly, peripheral blood samples were collected, centrifuged to obtain serum, and stored at −80 °C until analysis. All samples were analyzed following the completion of patient enrollment in May 2018. All samples were thawed only once prior to lipid extraction, and repeated freeze–thaw cycles were strictly avoided to preserve sample integrity. Lipid metabolite extraction was then performed. Ultra-high-performance liquid chromatography (UHPLC) was then used for detection, and XCMS was employed for data preprocessing and annotation to obtain the relative quantification of omega-3 fatty acids. In our study, the detected omega-3 fatty acids comprised DHA and EPA. The detection method used in this study yielded relative quantification of fatty acid concentrations rather than their absolute quantification. The detailed experimental procedures were described previously (Zhou et al., 2024).

### Statistical analysis

The baseline characteristics of patients who experienced ECOPD-related readmission were compared with those who did not. Based on the results, patients were further stratified into low-DHA and high-DHA groups according to the median DHA level. Statistical analyses were performed using Student’s *t*-test or the Mann–Whitney U test for continuous variables. Continuous variables were presented as mean ± standard deviation (SD) for normally distributed data or as median with interquartile range (25th–75th percentile) for non-normally distributed data. Categorical variables were compared using the Chi-square test or Fisher’s exact test and were reported as numbers and percentages. Event-free status was defined as the absence of ECOPD-related readmission during the follow-up period. Kaplan–Meier survival curves and log-rank tests were used to compare the clinical outcomes between the low- and high-DHA groups. Additionally, univariable and multivariable Cox proportional hazards regression models were performed to identify risk factors influencing event-free time. The results were reported as hazard ratios (HRs) with 95% confidence intervals (CIs). Variables with *P* < 0.1 in univariable analysis or those considered clinically relevant were included in the multivariable model for further analysis. A nomogram was constructed based on identified risk factors, and a calibration curve was used to assess the model’s predictive performance. To explore the relationship between DHA levels and systemic inflammation, Spearman’s rank correlation analysis was performed between baseline serum DHA concentrations and inflammatory markers. All statistical analyses were conducted using R software (version 4.3.1; R Core Team, 2023). A two-sided *P*-value <0.05 was considered statistically significant. All data were analyzed anonymously.

## Results

### Baseline characteristics

**Figure 1:**
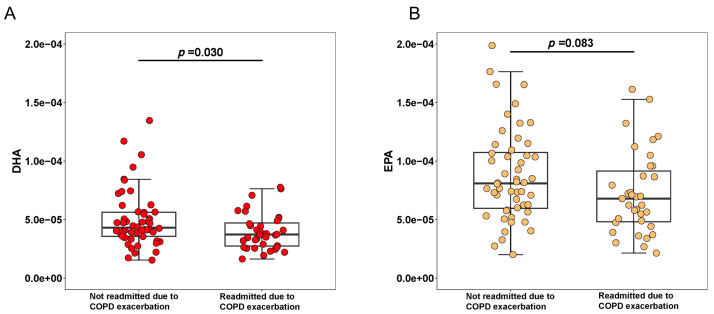
Box plots comparing the levels of DHA (A), EPA (B) between patients with and without COPD-related readmissions.

Among the 88 patients included in our study, 85.23% were male, with a mean age of 73.66 years. The baseline distribution of the cohort according to the GOLD classification is presented. Based on post-bronchodilator FEV_1_ % predicted, 95.3% of patients were classified as GOLD stages II–IV, with 26.6% in GOLD stage II, 42.2% in GOLD stage III, and 26.6% in GOLD stage IV. During a median follow-up period, 36 patients (40.91%) experienced ECOPD-related readmission. There were no significant differences between patients with and without ECOPD-related readmission in terms of age, sex, body mass index (BMI), spirometry function, or inflammatory markers such as CRP, procalcitonin (PCT). Patients who experienced ECOPD-related readmission had a longer hospital stay compared to those who did not, though the difference was not statistically significant. These patients also had a higher prevalence of comorbid heart failure and elevated fibrinogen levels. The proportion of patients receiving systemic corticosteroids during the current hospitalization was similar between the two groups. Additionally, we compared maintenance treatments after discharge from the index hospitalization between the two groups, and no significant differences were found in the use of inhaled corticosteroids (ICS), long-acting beta-agonists (LABA), or long-acting muscarinic antagonists (LAMA) (Table 1). Regarding fatty acid levels, DHA levels were significantly lower in patients who experienced ECOPD-related readmission. EPA exhibited no significant differences between the two groups (Fig. 1).

**Table 1:** Demographic, clinical characteristics, fatty acid levels of the study subjects.

	**Overall (*n* = 88)**	**Readmitted due to COPD exacerbation (*n* = 36)**	**No readmitted due to COPD exacerbation (*n* = 52)**	***p.*** **value**
**Male**	75 (85.23%)	33 (91.67%)	42 (80.77%)	0.267
**Age**	73.66 (9.12)	74.22 (9.40)	73.27 (9.00)	0.636
**BMI**	22.47 (4.40)	22.51 (3.83)	22.44 (4.79)	0.940
**Packyear**	38.00 (18.75, 60.00)	40.00 (24.50, 60.00)	30.00 (10.00, 60.00)	0.176
**FEV1/FVC**	49.82 (9.85)	48.23 (9.00)	50.92 (10.34)	0.199
**FEV1%predicted**	41.00 (33.50, 48.00)	41.00 (31.23, 41.25)	41.00 (36.00, 51.40)	0.122
**Hypercapnic respiratory failure**	23 (26.14%)	6 (16.67%)	17 (32.69%)	0.150
**Number of hospitalizations for COPD exacerbation in the past year ≥ 1**	31 (35.23%)	16 (44.44%)	15 (28.85%)	0.201
**Comorbidities**				
Hypertension	49 (55.68%)	20 (55.56%)	29 (55.77%)	1.000
Coronary heart disease	17 (19.32%)	6 (16.67%)	11 (21.15%)	0.803
Heart failure	8 (9.09%)	0 (0.00%)	8 (15.38%)	0.019
Diabetes	13 (14.77%)	3 (8.33%)	10 (19.23%)	0.267
Hyperlipidemia	9 (10.23%)	3 (8.33%)	6 (11.54%)	0.732
Sleep apnea	2 (2.27%)	1 (2.78%)	1 (1.92%)	1.000
**ACCI**	5.00 (4.00, 6.00)	4.50 (3.75, 5.00)	5.00 (4.00, 6.00)	0.550
Eosinophil count (*10^9^/L)	0.04 (0.00, 0.13)	0.03 (0.00, 0.11)	0.05 (0.00, 0.14)	0.568
**Eosinophil percent (%)**	0.65 (0.08, 2.17)	0.45 (0.08, 1.62)	0.70 (0.08, 2.40)	0.575
**CRP (mg/dL)**	1.31 (0.95, 2.06)	1.31 (1.16, 3.29)	1.31 (0.60, 1.95)	0.267
**D-dimer (*μ*g/mL)**	0.20 (0.13, 0.35)	0.20 (0.15, 0.33)	0.20 (0.12, 0.38)	0.687
**Fibrinogen (g/L)**	3.68 (3.08, 4.36)	4.06 (3.41, 4.61)	3.45 (3.02, 4.29)	0.043
**PCT (ng/mL)**	0.06 (0.04, 0.13)	0.06 (0.05, 0.09)	0.06 (0.04, 0.14)	0.706
**BNP (pg/mL)**	223.70 (98.62, 537.75)	223.70 (65.66, 488.95)	223.70 (127.47, 568.55)	0.304
**In-hospital systemic hormone therapy**	40 (40.45%)	18 (50.00%)	22 (42.31%)	0.621
**Length of hospital stay(days)**	13.00 (9.00, 16.00)	14.00 (11.75, 19.25)	12.00 (8.00, 16.00)	0.056
**Maintenance therapy after discharge**				
ICS	57 (64.77%)	26 (72.22%)	31 (59.62%)	0.322
LABA	55 (62.50%)	25 (69.44%)	30 (57.69%)	0.370
LAMA	68 (77.27%)	28 (77.78%)	40 (76.92%)	1.000
**Fatty acids (relative concentration)**				
DHA	4.097e−05 (3.252e−05, 5.135e−05)	3.724e−05 (2.738e−05, 4.711e−05)	4.312e−05 (3.573e−05, 5.625e−05)	0.030
EPA	7.374e−05 (5.605e−05, 1.048e−04)	6.868e−05 (4.856e−05, 9.585e−05)	8.093e−05 (5.961e−05, 1.073e−04)	0.083

**Notes.**

Data are presented as mean (SD), median (25th percentile, 75th percentile) or n (%). ICS, inhaled corticosteroids; LABA, long-acting beta-agonists; LAMA, long-acting muscarinic antagonists; FEV1, Forced Expiratory Volume in 1 s; FVC, Forced Vital Capacity; ACCI, Age-Adjusted Charlson Comorbidity Index; CRP, C-Reactive Protein; PCT, Procalcitonin; DHA, Docosahexaenoic Acid; EPA, Eicosapentaenoic Acid.

### Kaplan–Meier analysis based on DHA levels

**Figure 2:**
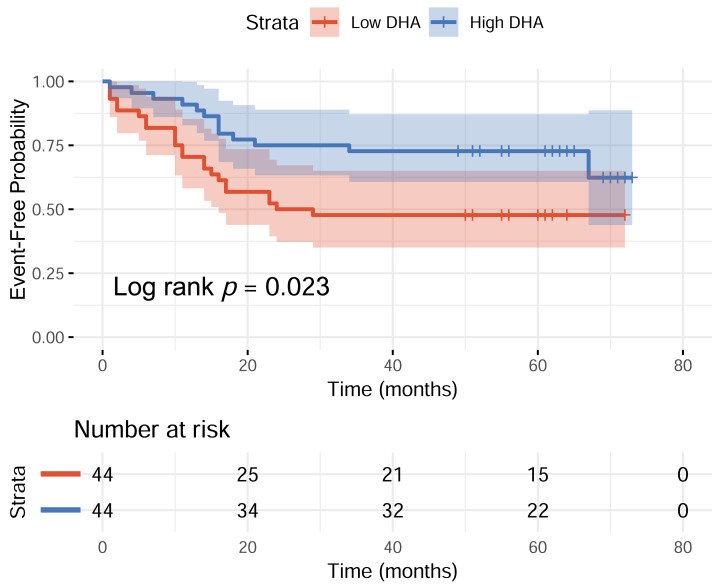
Kaplan–Meier curve of event-free probability according to DHA levels (Low *vs* High DHA).

The median normalized peak intensity value for DHA among all participants was 4.097 × 10^−^^5^. Based on this median value, participants were categorized into low-DHA and high-DHA groups. During the follow-up period, the proportions of participants who experienced acute exacerbation-related readmission were 52.27% in the low-DHA group and 29.55% in the high-DHA group. The Kaplan–Meier curve depicted the probability of remaining event-free from ECOPD-related hospitalization among patients with low- and high-DHA levels. During the overall follow-up period, patients in the Low-DHA group had a significantly higher ECOPD-related readmission rate compared to those in the High-DHA group (log-rank *p* = 0.023) (Fig. 2). This suggested that low-DHA levels may be associated with an increased risk of ECOPD-related hospitalization in COPD patients.

### Risk factor analysis for overall ECOPD-related readmission

The univariable and multivariable Cox regression analysis results for overall ECOPD-related readmission are presented in Table 2. To assess the contribution of different factors to ECOPD-related readmission risk, we developed two multivariable Cox regression models. Model 1 was constructed using variables with *p* < 0.1 in univariable analysis. Model 2 was developed based on clinically relevant variables. In Model 1, low DHA levels (HR: 3.774; 95% CI [1.745–8.130]; *p* = 0.001) was identified as the independent risk factor for overall ECOPD-related readmission, while FEV_1_% predicted (HR: 0.971; 95% CI [0.944–1.000]; *p* = 0.047) was identified as an independent protective factor. In Model 2, low DHA levels (HR: 3.509; 95% CI [1.582–7.752]; *p* = 0.002) remained a significant independent risk factor, while FEV_1_% predicted (HR: 0.958; 95% CI [0.927–0.989]; *p* = 0.009) continued to act as an independent protective factor. CRP and PCT did not show a significant association with overall ECOPD-related readmission.

**Table 2:** Univariate and multivariate Cox regression analyses for the hospitalization incidence due to COPD exacerbations.

	**Univariable**		**Multivariable -Model 1**		**Multivariable-Model 2**	
	Hazard ratio (95% CI)	*P* value	Hazard ratio (95% CI)	*P* value	Hazard ratio (95% CI)	*P* value
Low DHA	2.208 [1.117, 4.367]	0.023	3.774 [1.745, 8.130]	0.001	3.509 [1.582, 7.752]	0.002
Male	2.291 [0.702, 7.474]	0.169			1.622 [0.462, 5.693]	0.450
Age	1.006 [0.970, 1.044]	0.748			1.023 [0.975, 1.073]	0.355
BMI	0.988 [0.920, 1.061]	0.746			1.040 [0.960, 1.127]	0.355
Pack-year	1.004 [0.994, 1.014]	0.434				
Hypercapnic respiratory failure	0.636 [0.247, 1.637]	0.348				
Number of hospitalizations for COPD exacerbation in the past year≥ 1	1.741 [0.902, 3.363]	0.099	1.392 [0.710, 2.729]	0.336	1.674 [0.828, 3.381]	0.151
Length of hospital stay (days)	1.044 [0.999, 1.091]	0.056	1.037 [0.984, 1.092]	0.174	1.927 [0.361, 10.282]	0.443
ACCI ≥ 4	1.706 [0.409, 7.112]	0.463				
FEV1/FVC	0.979 [0.946, 1.013]	0.228				
FEV1%Predicted	0.979 [0.957, 1.003]	0.082	0.971 [0.944, 1.000]	0.047	0.958 [0.927, 0.989]	0.009
EOS ≥ 0.2 ×10^9^/L	0.796 [0.310, 2.048]	0.636				
EOS% ≥ 2%	0.663 [0.302, 1.457]	0.307				
CRP	1.040 [0.970, 1.114]	0.270				
D-dimer	0.608 [0.145, 2.552]	0.496				
Fibrinogen	1.226 [0.985, 1.526]	0.069	1.256 [0.986, 1.601]	0.065		
PCT	0.055 [0.001, 2.036]	0.115				
BNP	1.000 [1.000, 1.000]	0.322				

**Notes.**

BMI Body Mass Index ACCI Age-Adjusted Charlson Comorbidity Index EOS Eosinophils CRP C-Reactive Protein PCT Procalcitonin BNP B-type Natriuretic Peptide FEV1 Forced Expiratory Volume in 1 s FVC Forced Vital Capacity.

### Nomogram for survival prediction based on DHA

**Figure 3:**
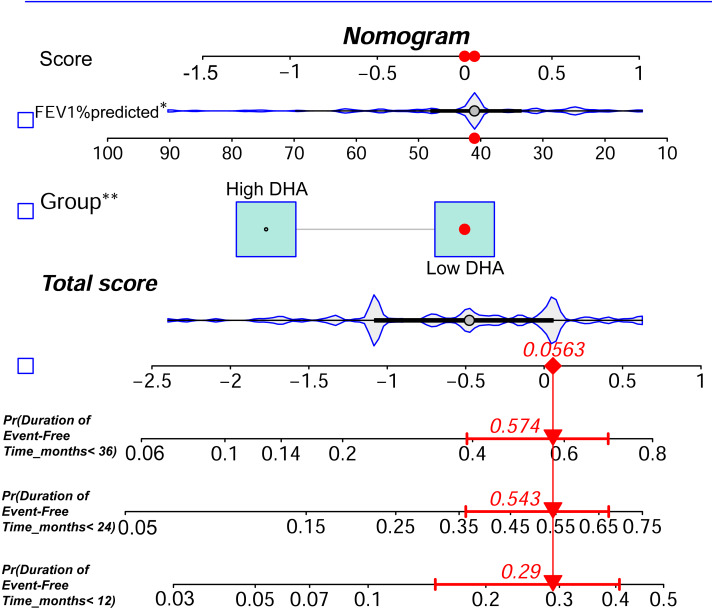
DHA-based nomogram for predicting long-term event-free status in patients with exacerbation-prone COPD.

**Figure 4:**
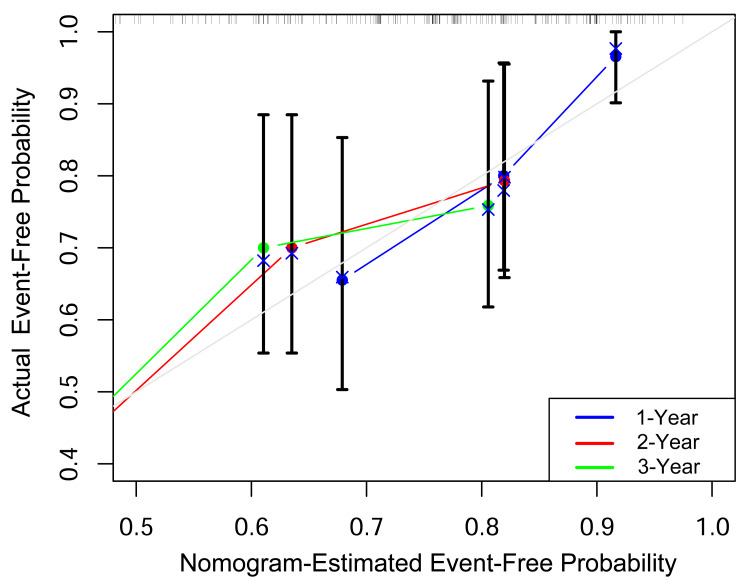
Calibration curves assessing the nomogram’s performance in predicting 1-year, 2-year and 3-year event-free status.

Based on the multivariable analysis results from Model 1, we constructed a nomogram that integrated Low DHA levels and FEV_1_% predicted to predict the probability of remaining event-free from ECOPD-related hospitalization in COPD patients at 1, 2, and 3 years (Fig. 3). To evaluate the predictive accuracy of the nomogram, we constructed a calibration curve. As shown in Fig. 4, the calibration curve demonstrated a good fit, indicating that the nomogram had robust predictive performance. This suggests that the model could serve as a valuable prognostic tool for patients with COPD.

### Association between DHA and systemic inflammation

**Figure 5:**
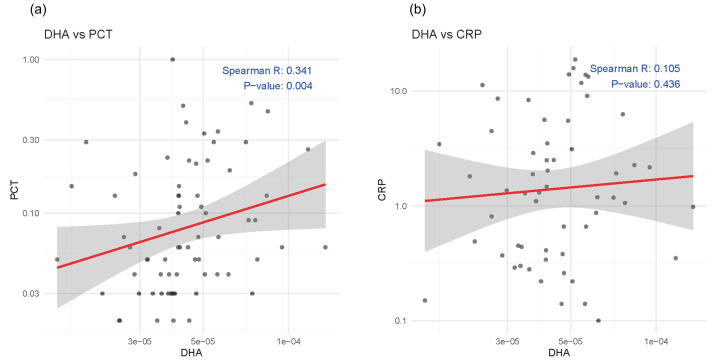
Scatter plots showing the correlations between serum DHA and inflammatory markers: (A) procalcitonin (PCT) and (B) C-reactive protein (CRP).

To explore the association between baseline serum DHA levels and systemic inflammation, we conducted Spearman’s rank correlation analyses with two commonly used inflammatory markers PCT and CRP. As shown in the scatter plots (Fig. 5), DHA was positively correlated with PCT (*p* = 0.004), while no significant correlation was found between DHA and CRP.

## Discussion

The highlight of this study was to explore the relationship between serum omega-3 fatty acid levels and the risk of ECOPD. In our retrospective cohort study, we observed that patients with lower DHA levels had a higher likelihood of being readmitted due to ECOPD, which was confirmed by the multivariate analysis models. Based on these insights, we developed a nomogram model to assess how well DHA levels can predict the risk of future ECOPD-related readmissions in these patients.

DHA, one of the main members of omega-3 fatty acid family, is known for its anti-inflammatory properties. Previous studies have demonstrated that increased omega-3 fatty acid intake can reduce the risk of COPD development (Shahar et al., 2008; Varraso et al., 2015). Moreover, higher omega-3 fatty acid intake has been associated with lower TNF-α concentrations in COPD patients (De Batlle et al., 2012), indicating its systemic anti-inflammatory effect. In addition, studies have demonstrated that serum and erythrocyte membrane omega-3 fatty acid levels were generally reduced in COPD patients (Gangopadhyay, Vijayan & Bansal, 2012; Novgorodtseva et al., 2013; Titz et al., 2016), resulting in decreased cell membrane fluidity and function impairment. Higher omega-3 fatty acid levels in peripheral blood were associated with better lung function and a reduced risk of airway damage (Kompauer et al., 2008; Shahar et al., 1999; Xu et al., 2019). And a large longitudinal study (*n* = 15,063) further revealed that higher DHA levels were positively associated with a slower rate of lung function decline (Patchen et al., 2023). Moreover, several clinical studies on acute lung injury have demonstrated that omega-3 fatty acid supplementation can effectively reduce pulmonary inflammation and promote the recovery of respiratory function (Huang et al., 2020). Animal studies have further supported the above findings, demonstrating that dietary or pharmacological supplementation of omega-3 fatty acids can prevent experimentally induced airway inflammation and accelerate lung tissue repair (Ulu et al., 2021).

Specialized pro-resolving lipid mediators derived from DHA, including Resolvin D1 (RvD1), Resolvin D2 (RvD2), and Maresin-1 (MaR1), have been confirmed to exhibit anti-inflammatory properties. In an animal model induced by chronic agricultural dust exposure, mice fed a high-DHA diet exhibited significantly increased levels of RvDs in bronchoalveolar lavage fluid (BALF), accompanied by reduced levels of various cytokines and chemokines, as well as decreased inflammatory cell infiltration and lymphoid aggregate formation in lung tissue (Ulu et al., 2021). A recent study showed that lower levels of RvD1 were associated with persistent respiratory symptoms after COPD exacerbation. Furthermore, exogenous RvD1 significantly reduced IL-6 and CXCL8 responses in COPD bronchial epithelial cells following rhinovirus infection(Finney et al., 2025). RvD1 was shown to mitigate smoking-induced emphysema by inhibiting inflammatory responses and oxidative stress in mice exposed long-term to cigarette smoke (Hsiao et al., 2015). Additionally, RvD1 reduced eosinophils and neutrophils and interleukin-6 (IL-6) levels in BALF and attenuated lung destruction in emphysema animal models (Kim et al., 2016). In another study, RvD1 was found to accelerate the resolution of lung inflammation by promoting alternative activation of macrophages to the M2 subtype and enhancing neutrophil phagocytosis (Hsiao et al., 2013). RvD1 treatment was able to reduce mortality, improve lung pathology, and suppress LPS-induced neutrophil and monocyte recruitment, as well as inhibit TNF-α and IL-6 production in acute lung injury mouse model (Zhang et al., 2019). Similarly, MaR1, another DHA-derived anti-inflammatory mediator, was shown to reduce airway inflammation by decreasing neutrophil infiltration, suppressing IL-6, TNF-α, and IL-8 levels, and downregulating ICAM-1 expression (Nordgren et al., 2015). All the above results indicated the protective role of DHA and its derivatives against the development of COPD.

DHA may influence the fate and function of T lymphocytes through multiple mechanisms, thereby promoting inflammation resolution and reducing tissue damage. An *in vitro* study demonstrated that DHA can interfere with the migratory capacity of CD4^+^ T cells, affecting their ability to reach target tissues, partially elucidating the anti-inflammatory mechanism of omega-3 fatty acids (Cucchi et al., 2020). Additionally, Resolvin D1, Resolvin D2, and Maresin-1 have been shown to inhibit the differentiation of naïve CD4^+^ T cells into Th1 and Th17 subsets while also reducing cytokine secretion from Th1 and Th17 cells (Chiurchiù et al., 2016). In various disease models, including sepsis-induced acute lung injury, experimental autoimmune encephalomyelitis, and psoriasis, exogenous omega-3 fatty acids such as DHA have been shown to suppress Th17 differentiation and the expression of related inflammatory mediators (Han et al., 2015; Kong, Yen & Ganea, 2011; Qin et al., 2014; Shoda et al., 2015; Xia et al., 2020).

Interestingly, baseline serum DHA levels were found to be positively correlated with PCT, this positive association may reflect a compensatory or reactive increase in DHA mobilization during systemic inflammation, possibly as part of an endogenous resolution mechanism. This interpretation was supported by recent evidence showing that levels of resolvin D1 (RvD1), a specialized pro-resolving mediator derived from DHA, were positively correlated with inflammatory cytokines such as CXCL10, IL-6, and MPO in sputum samples during the first and second weeks after ECOPD (Finney et al., 2025). Taken together, these observations underscore the complexity of the inflammatory response in COPD. Future longitudinal cohort studies and mechanistic experiments are warranted to determine whether DHA levels are associated with the inflammation resolution and clinical recovery following exacerbation.

Overall, the anti-inflammatory effects of omega-3 fatty acids in the lungs are well established. Our findings indicate that low baseline serum DHA levels are significantly associated with an increased risk of ECOPD-related hospital readmission, highlighting the potential anti-inflammatory and pro-resolving roles of DHA in this context. This observation supports the utility of DHA as a biomarker for individualized risk stratification in COPD patients. Given the variability in DHA levels across individuals, baseline assessment may help identify patients at heightened risk of poor outcomes following exacerbation. Importantly, the association between low DHA and increased readmission risk remained significant after adjusting for lung function and systemic inflammatory markers, suggesting that DHA may serve not only as a prognostic biomarker but also as a modifiable therapeutic target. Prior studies have demonstrated that DHA and its derivatives, such as resolvin D1 and maresin-1, promote the resolution of airway inflammation, further supporting its biological plausibility. Building on this rationale, interventional trials are warranted to evaluate whether DHA supplementation can reduce exacerbation-related readmissions, particularly in patients with low baseline DHA levels. Taken together, these findings provide a strong rationale for integrating DHA assessment into precision medicine strategies for COPD, both in risk prediction and therapeutic planning.

Furthermore, the potential influence of comorbidities on serum DHA levels required careful consideration. Common comorbidities in COPD, such as heart failure, malnutrition, and metabolic disorders, may affect circulating omega-3 fatty acid concentrations and thereby confound the observed associations. To minimize this potential bias, we excluded patients with active malignancy or end-stage organ dysfunction. Additionally, we adjusted for overall comorbidity burden using the age-adjusted Charlson Comorbidity Index (ACCI) and included BMI in the multivariable models as a surrogate marker of nutritional status. Importantly, DHA remained a significant independent predictor of ECOPD-related readmission after these adjustments.

This study had several limitations. First, as a single-center retrospective study, selection bias cannot be entirely avoided. To strengthen external validity, future research should include multicenter prospective cohorts. Second, the sample size was relatively small, and although a calibration curve was used to assess the nomogram’s performance, an independent validation cohort was not available. Therefore, large-scale, multi-center, prospective studies were needed to further validate our findings. Third, we did not collect detailed information on dietary omega-3 fatty acid intake or supplement use. Therefore, residual confounding due to unmeasured lifestyle or nutritional factors cannot be excluded. Incorporating standardized dietary assessments could address this in future work. Fourthly, the DHA levels in this study were obtained as relatively quantitative data using LC-MS. In our future research, an optimal absolute quantification threshold for DHA would be established to more accurately assess its relationship with the risk of COPD exacerbation. Fifthly, specific factors such as malnutrition and sarcopenia were not directly assessed in our study and should be comprehensively evaluated in future studies. Lastly, this study did not incorporate multidimensional indices such as the BODEX (body mass index, airflow obstruction, dyspnea, and previous severe exacerbations) indices. Future research could integrate these indices to enhance risk stratification and improve comparability with other studies.

## Conclusion

This study demonstrates that low baseline serum DHA level is an independent and reliable predictor of ECOPD-related hospital readmission in patients with COPD. While previous research has established the anti-inflammatory and protective properties of DHA in stable COPD, our findings extend this knowledge by highlighting its prognostic relevance during the post-exacerbation period. This novel association underscores the potential utility of serum DHA as a biomarker for individualized risk stratification in clinical practice. Given the growing burden of COPD exacerbations, incorporating DHA assessment into routine management may help identify high-risk patients who could benefit from targeted interventions. Future prospective and interventional studies are warranted to determine whether DHA supplementation can reduce the risk of recurrent exacerbations and improve long-term outcomes.

## Supplemental Information

10.7717/peerj.20865/supp-1 Supplemental Information 1 Code

10.7717/peerj.20865/supp-2 Supplemental Information 2 Sample Size Estimation

10.7717/peerj.20865/supp-3 Supplemental Information 3 Data

10.7717/peerj.20865/supp-4 Supplemental Information 4 Codebook

10.7717/peerj.20865/supp-5 Supplemental Information 5 STROBE checklist

## Additional Information and Declarations

### Data Availability

The following information was supplied regarding data availability: The data is available at figshare: Zhou, Qiqiang (2025). NEG-Mean.xlsx. figshare. Dataset. https://doi.org/10.6084/m9.figshare.30128149.v2.
