# Supplementary material for: Serum docosahexaenoic acid as a predictor of hospital readmission in chronic obstructive pulmonary disease exacerbation: a retrospective cohort study
Source: PeerJ. 2026 Feb 19;14:e20865. doi: 10.7717/peerj.20865 (PMC12925419; doi:10.7717/peerj.20865)
Supplement: Supplemental Information 2 [file peerj-14-20865-s002.pdf]

## Supplemental Articles 1. Sample Size Estimation

A post hoc power analysis was performed using PASS software (PASS 2025, version 25.0.2.) to evaluate whether the sample size was sufficient to detect the observed association between DHA levels and readmission. Based on multivariable Cox model 1, the hazard ratio (HR) for low DHA levels was 3.774, corresponding to a log hazard ratio (β) of 1.329. The event rate in the cohort was 40.9%, and the standard deviation of the binary DHA variable was 0.5. The R-squared between the low DHA indicator and other covariates included in the model was calculated to be 0.08. Under these parameters, the minimum sample size required to achieve 90% power at a two-sided α level of 0.05 was estimated to be 64. Since our study included 88 patients, the sample size was considered adequate for detecting the observed effect.
